# Metastasis in the wild: investigating metastasis in non-laboratory animals

**DOI:** 10.1007/s10585-019-09956-3

**Published:** 2019-02-09

**Authors:** Bushra Abu-Helil, Louise van der Weyden

**Affiliations:** Experimental Cancer Genetics (T113), Wellcome Sanger Institute, Wellcome Genome Campus, Hinxton, Cambridge, CB10 1SA UK

**Keywords:** Metastasis, Cancer, Animal model, Veterinary, Domestic, Free-living, Comparative oncology

## Abstract

Humans are not the only species to spontaneously develop metastatic cancer as cases of metastasis have been reported in a wide range of animals, including dinosaurs. Mouse models have been an invaluable tool in experimental and clinical metastasis research, with the use of genetically-engineered mouse models that spontaneously develop metastasis or ectopic/orthotopic transplantation of tumour cells to wildtype or immunodeficient mice being responsible for many key advances in our understanding of metastasis. However, are there other species that can also be relevant models? Similarities to humans in terms of environmental exposures, life-span, genetics, histopathology and available therapeutics are all factors that can be considered when looking at species other than the laboratory mouse. This review will explore the occurrence of metastasis in multiple species from a variety of domestic, captive and free-living veterinary cases to assist in identifying potential alternative experimental and clinical research models relevant to humans.

## Introduction

Metastasis continues to be the main cause of mortality in human cancers. Despite infrequent development of spontaneous tumours in wild rodent populations [1], mice are the most commonly used model in cancer research and continue to be effective, however are limited in recapitulating the complexity of the metastatic cascade, with a < 8% successful translation rate from animal models to clinical cancer trials [2]. Utilising the spontaneously occurring instances of metastasis outside of inbred controlled laboratory populations may be the way to obtain more relevant and faithful models. Recent advancements in the detection, treatment and monitoring of cancer progression has contributed to an increase of veterinary pathology cases reporting metastasis. In addition, veterinarians find that for companion animals, there is generally a very high rate of compliance with treatment and re-check visits, and an 80–90% rate of necropsy (autopsy) [3]. Confirmation of malignancy in domestic animals is via histological examination, and the World Health Organisation’s (WHO) grading and TNM staging system specifically for domestic animals continues to be commonly used in veterinary reports to characterise disease progression in an internationally recognised and standardised manner [4]. In many of these cases, cytokeratin markers and altered protein expression levels are used to differentiate metastatic nodules, analogous to that performed in the diagnosis of metastasis in human patients [5, 6]. Domestic, in particular companion animals, are susceptible to disease states present in some humans (such as obesity, diabetes, stress), and exposed to similar environmental factors that humans encounter (such as pollution, second-hand smoke), thus they represent relevant models to investigate the roles these parameters play in both the development of metastasis and efficacy of its treatment. Even in non-domestic species there are detailed veterinary reports of metastasis occurrences in a wide range of animals. The use of non-domestic species affords the opportunity to study the natural history of metastasis development without the confounding effects of therapy, which is rarely achievable in human studies. Thus, there is much we could learn about metastasis from broadening our focus of experimental models beyond laboratory-bred animals.

## Occurrences of spontaneous metastasis in non-laboratory animals

Whilst it is not surprising that metastasis would occur in non-laboratory animals belonging to the *Mammalia* class (examples listed in Table 1), extensive searching of the literature has shown evidence of spontaneous metastases in an amazingly wide range of non-mammalian species. For example, marine bivalves, such as the softshell clam (*Mya arenaria*) can develop germinomas (gonadal neoplasms) that are capable of metastasizing. In a study of softshell clams from Long Cove in Maine between 1971 and 1975, up to 22% of individuals examined had neoplasms and metastasis was noted in 40% of these cases [7]. Twelve fully grown pike (*Esox lucius*) kept in a tank at the New York Aquarium all died at various intervals between 1940 and 1941 and autopsies showed massive growths in the kidneys (histopathologically diagnosed as lymphosarcomas), with metastasis to the spleen and liver [8]. A retrospective survey of neoplasia in Philadelphia Zoo from 1901 to 2002 revealed 5/19 (25%) of the neoplasms found in lizards and 6/58 (9%) of the neoplasms found in snakes, all of which were carcinomas, showed evidence of metastasis (primarily to the liver, but also the pancreas, lung, spleen, and mesentery) [9]. Metastatic squamous cell carcinoma spreading to the liver, lung and/or kidneys has been described in case reports of two stranded loggerhead sea turtles (*Caretta caretta*) [10] and a wild-caught saltwater crocodile (*Crocodylus porosus*) [11]. There is a case report of a cockatiel (*Nymphicus hollandicus*) with a seminoma that had metastasized to the liver [12]. Even a section of *Allosaurus* humerous from the Upper Jurassic Morrison Formation (collected in Colorado) showed evidence of the presence of metastatic cancer [13]. Additional examples of non-mammalian species with reports of metastatic cancer are listed in Table 2.

**Table 1:** Examples of metastases in mammalian species

Infraclass	Order	Family	Common name	Latin name	Cancer type	Metastasis site(s)
Eutherians/Placental	Carnivora	Felidae	Lion	*Panthera leo*	Scirrhous solid mammary carcinomas	Regional lymph nodes [14]
			Ocelot	*Leopardus pardalis*	Uterine transitional cell carcinoma	Lumbar vertebrate [15]
		Hyaenidae	Spotted hyena	*Crocuta crocuta*	Squamous cell carcinoma	Multiple lymph nodes, lungs, adrenal glands, mesentry, adipose tissue, pelvic canal and skeletal muscle [16]
		Mustelidae	Sea otter	*Enhydra lutris*	Cholangiocellular carcinoma Malignant seminoma	Widespread [5] Regional lymph nodes [5]
			Black footed-ferret	*Mustela nigripes*	Mammary papillary cystadenocarcinoma	Regional lymph nodes, lungs, liver, spleen and mesentery [17]
		Otariidae	Californian sea lion	*Zalophus californianus*	Metastatic carcinoma	Regional lymph nodes, penis, prepuce, vagina, cervix and uterus [18] Lung, spleen, liver, kidney, omentum and mesentery LN [19]
		Ursidae	Polar bear	*Ursus maritimus*	Osteolytic osteosarcoma	Pulmonary [20]
			Eurasian brown bear	*Ursus arctos arctos*	Ovarian carcinoma	Regional lymph nodes and brain [21]
			Himalyan brown bear	*Ursus arctos*	Mammary papillary cystadenocarcinoma	Regional lymph nodes, liver and intestine [22]
			Giant panda	*Ailuropoda melanoleuca*	Ovarian granulosa cell tumour	Lungs, spleen, kidneys and perianal tissue [23]
	Artiodactyla	Bovidae	Addax	*Addax nasomaculatus*	T-cell cutaneous lymphoma	Multiple lymph nodes [24]
		Camelidae	Alpaca	*Vicugna pacos*	Mucosal melanoma Mammary adenocarcinoma	Regional lymph nodes, pulmonary parenchyma, pericardium and diaphragmatic parietal pleura [25] Regional lymph node, adrenal gland, liver, lung and spinal colon [26]
			Llama	*Lama glama*	Metastatic uterine adenocarcinomas Scirrhous anaplastic mammary adenocarcinoma Pulmonary adenocarcinoma	Pulmonary, hepatic parenchyma, plural and peritoneal surfaces [27] Regional lymph nodes, lungs, rectum, adrenal glands, cutaneous tissues and liver [26] Lungs and liver [28]
		Giraffidae	Rothschild’s Giraffe	*Giraffa camelopardalis rothschildi*	Embryonal rhabdomyosarcoma	Regional lymph nodes, lungs and sinuses [29]
		Monodontidae	Beluga whale	*Delphinapterus leucas*	Mammary adenocarcinoma Adenocarcinoma of salivary gland	Regional lymph nodes, liver, lung, spleen, adrenal gland, ovary, bone marrow of humerus and ribs [30–32]
		Delphinidae	Atlantic bottlenose dolphin	*Tursiops truncatus*	Uterine adenocarcinoma Malignant seminoma	Lungs, lymphatic vessels or diaphragm, walls and veins of all organs [33] Intestine, mesenteric lymph nodes, kidney, pancreas, liver, and spleen [34]
			Atlantic spotted dolphin	*Stenella frontalis*	Uterine T-cell lymphoma Malignant seminoma	Gastric compartment, peritoneum, lung parenchyma, serosa of the thoracic aorta, pituitary gland and adrenocortical layers [34] Retroperitoneal area and adjacent muscle [35]
		Phocoenidae	Harbour porpoise	*Phocoena phocoena*	Gastric adenocarcinoma	Peritoneal cavity, uterus, lymph nodes and liver [36]
	Eulipotyphla	Erinaceidae	African pygmy hedgehog	*Atelerix albiventris*	Amelanotic melanoma Mast cell tumour	Spleen and liver [6] Regional lymph nodes [37]
	Scandentia	Tupaiidae	Tree shrew	*Tupaia spp*	Malignant lymphoma Hepatocellular carcinoma Carcinoid pancreatic tumour	Liver, spleen and kidneys [38] Lungs [38] Lungs and adrenal glands [38]
	Rodentia	Caviidae	Capybara	*Hydrochoerus hydrochaeris*	Squamous cell carcinoma	Regional lymph nodes [39]
Metatheria/Marsupial	Dasyuromorphia	Dasyuridae	Tasmaninan devil	*Sarcophilus harrisii*	Devil facial tumour disease	Regional lymph nodes, lungs, spleen, heart, kidneys, ovary, serosal surface of the rib, mammary, adrenal and puitary glands [40]
	Diprotodontia	Macropodidae	Red kangaroo	*Macropus rufus*	Lymphadenopathy Squamous cell carcinoma T-cell lymphosarcoma	Lungs and mammary glands [41] Regional lymph nodes [41] Pancreas, intestines, gall bladder, subcutis muscle, skeletal muscle and cardiac muscle [41]
		Petauridae	Sugar glider	*Petaurus breviceps*	Mammary carcinoma	Multiple lymph nodes and lungs [42]
	Didelphimorphia	Didelphidae	Opossum	*Didelphis virginiana*	Mast cell tumour Acute myeloid lukemia Hepatic hemagiosarcoma	Liver, pancreas, kidney, spleen and skin [43] Regional lymph nodes, liver, lungs, kidneys and spleen [43] Kidney, heart and skin [43]
Prototheria/Monotrome	Afrosoricida	Tenrecidae	Lesser Madagascar hedgehog tenrec	*Echinops telfairi*	Amelanotic melanoma	Liver and spleen [6]

**Table 2:** Examples of metastases in non-mammalian species

Class	Order	Family	Common name	Latin name	Cancer type	Metastasis site(s)
Reptilia	Testudines	Cheloniidae	Loggerhead sea turtle	*Caretta caretta*	Squamous cell carcinoma	Pulmonary, ventricular mycocardium, skeletal muscle, liver, spleen, heart, lungs and kidney [10]
	Crocodilia	Crocodylidae	Salt water crocodile	*Crocodylus porosus*	Squamous cell carcinoma	Liver [11]
	Squamata (Serpentes)	Colubridae	Northern water snake	*Nerodia sipedon*	Splenopancreatic ductal adenocarcinoma	Widespread metastasis [44]
			Corn snake	*Elaphe guttata*	Renal cell carcinoma	Lung and liver [45]
			Western hognose snake	*Heterodon nasicus*	Ductal adenocarcinoma	Liver, kidney and intestine [46]
		Viperidae	Eastern diamondback rattlesnake	*Crotalus adamanteus*	Squamous cell carcinoma	Local vascular invasion [47]
Amphibia	Anura	Ranidae	Northern leopard frog	*Rana pipiens*	Lucké renal adenocarcinoma	Lung, liver, coelom, ovaries, pancreas, urinary bladder and spleen [48]
Actinopterygii	Esociformes	Esocidae	Northern pike	*Esox lucius*	Lymphosarcoma	Liver and Spleen [8]
Bivalvia	Myoida	Myidae	Soft-shell clam	*Mya arenaria*	Germinomas	Interfollicular connective tissue, body wall, epibranchial chamber, and genital ducts [7]
Aves	Sphenisciformes	Spheniscidae	African black-footed penguin	*Spheniscus demersus*	Cholangiocarcinoma	Lung, pancreas, mesentery and cloaca [49]
			Adelie penguin	*Pygoscelis adeliae*	Cloacolithiasis and intestinal lymphosarcoma	Liver and renal gland [50]
	Psittaciformes	Psittacidae	Macaw	*Ara* spp.	Oropharyngeal and cloacal papilloma	Liver, pancreas and intestines [51]
		Cacatuidae	Sulphur-crested cockatoo	*Cacatua galerita*	Ventriculus carcinoma	Lungs [52]
			Cockatiel	*Nymphicus hollandicus*	Bilateral Seminoma	Liver [12]
	Galliformes	Phasianidae	Plymouth-rock chicken	*Gallus gallus domesticus*	Osteoblastic osteocarcinoma	Liver [53]

However, whilst reports of metastases in animal species outside the laboratory is of great interest, an argument for the use of these animals as alternatives for experimental and clinical modelling of metastasis is only possible if their disease faithfully recapitulates the genetics, pathology and drug responses of that seen in humans. Despite the enormous range of species within the *Mammalia* class alone, there are many examples of striking similarities between metastasis in humans and other vertebrates. For example, embryonal rhabdomyosarcoma (ERMS) is the most common soft tissue sarcoma occurring in children. Typically occurring in the head and neck, ERMS is treated with surgical excision and adjuvant chemo- or radiation therapy, however survival of patients rapidly decreases with development of metastasis [54]. Mirroring this is a case report of a juvenile captive Rothschild’s giraffe with ERMS that was treated with surgical resection and 5-fluorouracil, however, it died shortly after due to the invasiveness of the disease and at time of necropsy there was evidence of metastatic spread to the sinuses, lymph nodes and lung [29]. Similarly, there is a case report of a 2 year old golden retriever with ERMS (in which diagnosis was confirmed by positive immunohistochemical staining for desmin, also used for diagnostic testing of ERMS in human patients) with metastatic spread to the lymph nodes and lungs, and despite three doses of radiation therapy and chemotherapy (vincristine, cyclophosphamide, and doxorubicin) it was eventually euthanized due to widespread metastatic disease [55]. Below we highlight further examples of similarities in the aetiology, histopathology, genetics and response to therapy of metastasis in non-laboratory bred mammals.

### Aetiological similarities

Breast cancer is the most common invasive cancer in women. The aetiology of invasive breast cancer in humans has been linked to a variety of causes, many of which are mirrored in the spontaneous development of aggressive mammary cancer (with metastasis) in animals. For example, epidemiological and experimental evidence implicates oestrogen exposure in the aetiology of breast cancer in humans (either from endogenous sources, hormone treatments or exposure environmental xenoestrogens (organochlorines)) [56] and is also considered a major factor in the development of aggressive mammary adenocarcinomas (including reports of metastasis) in the St. Laurence Estuary Beluga whale (*Delphinapterus leucas*) population [30]. The role of hormonal influence in the initiation and progression of breast cancer has also been recognised in canines [57]. Obesity increases the risk of breast cancer in post-menopausal women and is associated with a poor prognosis [58]. A study investigating the features of canine mammary carcinomas according to body score found a lower age of onset and more frequent lymphatic invasion of carcinoma cells in overweight or obese dogs compared with lean or ideal bodyweight dogs [59]. Obese women have been shown to have lower plasma adiponectin levels compared with non-obese women [60], and reduced adiponectin expression has been reported in mammary carcinomas from overweight or obese female dogs, with lymphatic invasion levels being lower in adiponectin-positive mammary carcinomas [59].

Approximately a fifth of all human cancers worldwide are caused by infectious agents; some examples of viruses causally linked to the aetiology of cancers include human papilloma virus in cervical cancer, hepatitis B virus in hepatocellular carcinoma, and herpesvirus [Epstein-Barr virus (human herpesvirus 4) and Kaposi’s sarcoma herpesvirus (human herpesvirus 8)] in Burkitt’s lymphoma and Kaposi’s sarcoma, respectively. All of these cancers are known to metastasize resulting in poor prognosis for the patient. Otarine herpesvirus-1 infection is a known cause of highly metastatic carcinomas of urogenital origin in free-living Californian sea lions (*Zalophus californianus*, as well as exposure to chemical and plastic waste contaminants in the water) [61]. In a study of Californian sea lions at a marine mammal rehabilitation centre (1979–1994), 76/370 (21%) of the animals had gross lesions on post-mortem examination, with 66/76 (18%) having widely metastatic carcinoma (subsequently determined to be of genital origin) [18]. Masses were found in the lungs and liver in 62% and 56% of the cases, respectively [19]. Ovine pulmonary carcinoma (OPC, also known as sheep pulmonary adenomatosis or jaagsiekte) is a retrovirus-induced bronchioloalveolar carcinoma [62] with morphological similarities to human bronchioloalveolar carcinoma. Metastases histologically resembling the primary tumor are found in bronchial or mediastinal lymph nodes in up to 10% of OPC cases, with metastases to other organs including the skeletal muscle, kidneys and lung occurring in 10–50% of cases, depending on the geographic location of the animals (reviewed in [63]). A recent case study reported progression of canine papillomavirus-associated cutaneous pigmented plaques to metastatic squamous cell carcinoma in two dogs [64].

Reports of spontaneous metastatic tumours in amphibians are uncommon with the majority of incidences being case studies of single animals [48]. However, an exception is the northern leopard frog (*Rana pipiens*), which is susceptible to Lucké tumour herpesvirus (LTHV)-induced renal adenocarcinoma [65, 66]. This tumour has the unique property of a metastatic phenotype that is regulated by the internal body temperature of the host. Whilst temperature exerts little effect on growth of the primary tumor, it profoundly affects the process of metastasis, with widespread metastatic colonies (lungs, liver, coelom, ovaries, pancreas, bladder, and other sites) being found in tumour-bearing frogs kept at 28 °C for 50 days, while those kept at 7 °C for 98 days or more have either no metastatic lesions or only an occasional small metastatic nodule [67]. Studies of this temperature-dependency metastatic phenotype have revealed that these tumors show increased motility and release of a metalloprotease (which degrades basement membrane collagen) at elevated temperatures [68].

Patterns in disease aetiology observed in marine species may contribute as an environmental biomarker as well as developing an understanding of carcinogen-induced metastasis. Chemical and plastic waste contaminants, such as polycyclic aromatic hydrocarbons (PAHs), polychlorinated biphenyls (PCBs) and organochlorines, are known human carcinogens. Their increasing presence in the oceans (due to human pollution) has been implicated in the development of urogenital carcinomas in Californian sea lions. The blubber of sea lions with genital carcinoma have 85% higher levels of PCBs than naïve controls [69]. Californian sea lions exhibit a high prevalence of metastatic carcinoma of urogenital origin (UGC) with 26% of adult sea lion post-mortems performed at The Marine Mammal Center in California (during 1998–2012) being affected [61]. Metastasis can occur both locally (abdominal and pelvic lymph nodes, kidney and urinary bladder) and to more distant sites (liver, lungs and spleen) [70].

The role of the immune system in cancer (from tumour initiation to metastatic progression) and how tumour cells avoid elimination by the immune attack is an area of current research [71], and whilst some cancers in animals have no direct human correlates per se, they can provide critical insights into tumour cell evasion of the immune system resulting in metastasis. For example, the clonally transmissible tumours that develop in and around the mouth, head and neck of Tasmanian devils (*Sarcophilus harrisii*, ‘devil facial tumour disease’, DFTD) are highly aggressive and frequently metastatic (59/91 cases), primarily spreading to regional lymph nodes and lungs [40]. One reason for the high fatality rate of this cancer is that Tasmanian devils have low genetic diversity (due to a prehistoric bottleneck and persecution from European settlers) and as such have a lack of diversity in most immunological genes, with all populations sharing similar MHC genes, so there is no immune response against the tumour cells [72]. Similar to DTFD, canine transmissible venereal sarcoma (CTVS) is a clonal cell allograft with highly metastatic potential. However, unlike the low diversity of MHC I in the Tasmanian devils, CTVS cells show downregulation of MHC I and a complete absence of MHC II, thus evading host immune recognition, permitting successful transmission and progression [73]. CTVS also causes severe damage to monocyte-derived dendritic cells, which has been proposed as another of its mechanisms for evading host immunity [74].

### Histopathological similarities

A lack of specific tools to appropriately utilise samples from spontaneous metastasis incidences outside of laboratory-controlled populations may be considered a limitation in investigating alternative models, however there are many successful reports of the diagnostic use of immunohistochemistry for biomarkers that are clinically relevant in the human setting [3]. For example, a cutaneous T cell lymphoma in an antelope (*Addax nasomaculatus*) with metastatic spread to multiple lymph nodes [24], an intestinal lymphosarcoma in an African black-footed penguin (*Spheniscus demersus*) with metastatic spread to the liver and kidneys [50], and a uterine T-cell lymphoma in an Atlantic bottle-nosed dolphin (*Tursiops truncates*) with metastatic spread to the lung, pituitary gland and peritoneum [35], all showed strong immunohistochemical staining for CD3 but not CD79a, confirming the lymphocytes to be of T cell origin (CD3 and CD79a are used as immunohistochemical markers of T and B-cell leukaemias/lymphomas in humans, respectively). In another case report, a cutaneous squamous cell carcinoma in a capybara (*Hydrochoerus hydrochaeris*) with regional lymph node metastases showed positive immunohistochemical staining for cytokeratin AE1/AE3, confirming the cell origin as being epithelial [39]. In addition, a pulmonary adenocarcinoma in a llama (*Lama glama*) with liver and bone metastases showed positive immunohistochemical staining for pan-cytokeratin, cytokeratin 7, and cytokeratin 5/6 antibodies and negative staining for vimentin and cytokeratins 8/18 and 20 antibodies, consistent with bronchioloalveolar carcinoma in humans [28].

In humans, metastatic ovarian cancer usually presents with widespread intra-abdominal metastasis remaining confined to the peritoneal cavity, however in cases of aggressive disease there can be lung, liver or central nervous system (CNS) metastases during disease progression. In an Eurasian brown bear (*Ursus arctos arctos*) with ovarian cancer, metastases were observed in the regional lymph nodes and brain, with immunohistochemistry showing strong positivity for cytokeratin AE1/AE3 [21], an antigen which is expressed by more than 90% of human ovarian carcinomas [75]. In a recent case report of a giant panda (*Ailuropoda melanoleuca*) with ovarian cancer, histopathological studies revealed multiple lesions in the lungs, kidneys, perianal tissue and spleen at necropsy, that stained positive by immunohistochemistry for B7-H4, CA125, and HE4, as well as the presence of significantly high serum levels of the tumour antigen AFP [23]. In humans, the combination of HE4 and CA125 expression is currently used in the clinic for accurate diagnosis of malignant ovarian tumours [76], and AFP is used in the clinic as a tumour marker.

Metastatic cutaneous melanoma in humans typically spreads to the lymph nodes, lungs, liver, bone and brain, and immunohistochemical markers commonly used for diagnosis include S-100, HMB-45, MART-1/Melan-A, tyrosinase, and MITF [77]. A case report of a lesser Madagascar hedgehog tenrec (*Echinops telfairi*) with amelanotic melanoma (presenting as a cutaneous mass on the ear), found extensive metastatic lesions in the spleen and liver, which were strongly HMB-45 positive and weakly S-100 positive by immunohistochemistry [6]. Another study of amelanotic melanomas in dogs showed strong immunohistochemical staining using rabbit anti-S100 in 26/31 of samples [78]. An uveal melanoma in a dog and its metastasis to the prostate, were also reported to show immunohistochemical positivity for Melan-A [79].

In humans, breast cancer typically spreads to the lymph nodes, bones, liver, lungs and brain. In wild animals there have been case reports of mammary adenocarcinomas in a lioness (*Panthera leo*) with spread to only regional lymph nodes [41] and a camel, llama and Beluga whale with widespread metastases [26, 30]. Interestingly, sections of the adenocarcinoma from the Beluga whale showed strong reactivity with a rabbit anti-human oestrogen receptor antibody [30]. Canine mammary tumour is the most common cancer among female dogs and often becomes fatal due to the development of distant metastases [80]. Metastasis to the regional lymph node is an early step in metastasis and is usually followed by the development of distant metastases, mainly in the lung, ultimately leading to the death of the dog [81, 82].

In dogs with osteosarcomas, > 95% present with pulmonary micrometastases [83], and in humans, metastatic osteosarcoma typically spreads to the lungs. There has also been a case report of a polar bear (*Ursus maritimus*) with osteosarcoma that was found to have multiple confluent metastases in all lobes of both lungs [20]. However, osteosarcoma can also spread to other organs such as the bone and brain, and although rare, there are case reports of osteosarcoma patients with liver metastases, as well as a report of an osteoblastic osteosarcoma in a free-range chicken (*Gallus gallus domesticus*) metastasizing to the liver [53].

The clinical course of tonsillar squamous cell carcinoma (SCC) in humans is aggressive with frequent recurrence and early dissemination (60–80% of patients have cervical lymph node metastasis at initial diagnosis [84]). SCC of tonsils in dogs are locally invasive and metastasize quite early to regional lymph nodes, with one study reporting 77% of their necropsy cases showing metastasis to regional lymph nodes or beyond [85]. Similarly, SCC of the tonsil in cats also tends to metastasize early to the regional lymph nodes [86].

Wilms’ tumour (nephroblastoma) typically occurs in children and often metastasizes, with those in stage IV (10% of cases, defined by the presence of hematogenous metastases to the lung, liver, bone, or brain) having a poor prognosis. Metastatic dissemination to distant sites has been reported in animals with nephroblastomas, including a Japanese eel (*Anguilla japonica*) [87] and a recent a case study of a miniature pinscher dog [88]. Interestingly, the histological appearance of metastatic lesions can be different from that of the primary tumour and in the case of the eel with nephroblastoma where hepatic metastases were found, the metastases differed markedly in histological type from the main primary tumour [87].

ERMS is the most common soft tissue sarcoma occurring in children. Desmin/myogenin/Myo-D1 immunohistochemical positivity is often used to confirm the diagnosis and treatment usually involves surgical excision and adjuvant chemo- or radiation therapy, however survival of patients rapidly decreases with development of metastasis [54]. Correspondingly, there is a necropsy report of an adolescent domestic canine with an invasive neoplasm in the cranial cavity and hepatic metastases, for which a diagnosis of ERMS was confirmed using immunohistochemical analysis for desmin and myogenin [89]. Similarly there is a case report of a young dog with eyelid enlargement (lachrymal gland protrusion) and multiple masses throughout the body, for which a diagnosis of ERMS was confirmed with immunohistochemical positivity of the tumour cells for desmin, myogenin and Myo-D1 [90].

In humans, renal cell carcinoma (RCC) has a great propensity for metastasis with 30% of patients already showing metastasis at the time of diagnosis, and metastatic RCC is a highly fatal disease [91]. Ninety percent of canine renal epithelial tumours are classified as malignant and metastases are detected in 50–60% of the cases [92]. In dogs, the most likely metastases sites are lungs, regional lymph node and liver, but occasionally also the brain and skin [92, 93], which is similar to that seen in humans [94]. Interestingly, metastases occur much more frequently in dogs than is reported in cattle with renal cell tumours [93, 95].

### Genetic similarities

Spontaneous tumours in non-laboratory animals are relevant models for human cancer primarily because both the animal population and the tumours themselves are genetically heterogeneous. As with metastatic breast cancer in humans, reduction and aberrant expression of BRCA1 in canine mammary tumours has been found to be significantly associated with malignant characteristics [96]. Similarly, altered derlin-1 and stanniocalcin-1 expression levels are associated with the metastasis of human breast cancer cells and metastisizing canine mammary adenocarcinomas [97]. Microarray analysis of 27 canine mammary carcinomas found 1,011 significantly differentially expressed genes between metastatic and non-metastatic carcinomas, and 265 of these genes were related to human breast cancer genes [98]. In addition, comparison with the van’t Veer 70 gene prognostic signature, which reliably identifies human breast cancers with metastatic potential, also found a significant overlap [99]. The claudins are tight junction proteins involved in cell adhesion and polarity, and studies have found reduced expression of claudins in human breast cancers [100], canine mammary carcinomas [101], and feline mammary cancers [102], is associated with a pre-invasion and metastasis phenotype. The human epidermal growth factor receptor 2 (HER2) is overexpressed in around 20–30% of breast cancers, and is associated with a more aggressive disease and increased mortality. Expression of the feline orthologue of the *HER2* gene, *f-HER2* (whose kinase domain is 92% similar to the *HER2* kinase domain), has been found to be increased in feline mammary carcinoma (FMC) cell lines and tissue samples, and the anti-human HER2 antibody strongly stained 13/36 (36%) FMC archival tissues samples (FMC is a highly aggressive, mainly hormone receptor–negative cancer, that been proposed as a model for poor prognosis triple negative breast cancer in humans) [103].

In human osteosarcoma, p53 has been shown to be an effective prognostic marker and upregulated p53 is associated with a shorter survival time [104], and this has also been associated with poor prognosis in canine osteosarcoma [105]. In addition to overall prognosis, genes such as the proto-oncogenic receptor *c-Met*, have been implicated in the development of metastasis in humans osteosarcoma [106], and in canine osteosarcoma, *c-Met* has been implicated in lymphatic spread [107]. The membrane cytoskeleton linking molecule, ezrin, has been associated with a shorted survival time (due to pulmonary metastasis) in both humans and dogs with osteosarcoma [108, 109].

Gastrointestinal stromal tumours (GISTs) are an aggressive cancer type that occurs in both humans and dogs [110], and have a poor prognosis due to the tumour commonly spreading to the liver and peritoneal cavity. In both species, the GISTs arise due to oncogenic mutations in the *KIT* tyrosine kinase, which also drives canine cutaneous mast cell tumours [111]. There are also case reports of a mast cell tumour in a llama, which exhibited positive membrane KIT expression [112], and a captive opossum (*Didelphis virginiana*), in which *c-KIT* positive cancerous cells were found to have spread to distant sites, including the liver, skin, kidney, pancreas and spleen [43].

Although there are conflicting reports of the presence of *BRAF, NRAS* and *c-KIT* mutations in canine mucosal melanomas, possibly reflecting the complexity and heterogeneity of cancer seen in humans, a strong parallel between human and canine mucosal melanoma is frequent in activation of the RAS/MAPK and/or PI3K/AKT/mTOR signalling pathways [57, 113] with synergistic targeted inhibition of MEK and dual PI3K/mTOR inhibiting tumour growth of a canine melanoma cell line in nu/nu athymic mice [114]. Recently, microarray expression profiling found upregulated expression of 8 miRNAs that could discriminate between metastasizing and non-metastasizing uveal melanomas in dogs, and 3 of these were found to be implicated as potential “metastasis activators” in human cutaneous melanoma [115].

### Therapeutic similarities

Immunotherapeutic targeting of immune checkpoint molecules (such as PD-1, PD-L1 and CTLA-4) have been widely used for some human cancers in the last 7 years, particularly melanoma, with Ipilimumab (CTLA-4 monoclonal antibody) treatment improving overall survival in patients with previously treated metastatic melanoma [116]. The expression of CTLA-4 and PD-1 has been observed on lymphocytes of dogs with mastocytoma, melanoma, and renal cell carcinoma, and studies using a human monoclonal antibody against PD-L1 has confirmed expression of PD-L1 on a number of canine tumour biopsies [117]. More recently, a cancer research report (2018) by the Flatcoated Retriever Society, which thanked the > 500 owners “who have kindly offered precious information for the good of the breed when grieving the loss of their beloved pet”, found that all their cases of histiocytic sarcomas (28 in total) showed expression of PD-L1. A pilot clinical trial to assess the clinical efficacy of a canine chimeric monoclonal antibody targeting PD-L1 in canine oral malignant melanoma or undifferentiated sarcoma showed a reduced tumour burden in some dogs, with the survival of dogs with metastatic oral malignant melanoma being prolonged in the antibody treatment group (*n* = 4) compared to an historical control group (*n* = 15) treated by standard therapies [118].

In humans, imatinib has been demonstrated to be highly effective in delaying progression and prolonging life in patients with metastatic GIST [119]. In a case report of a mixed-breed dog with GIST, five months after surgical resection of the tumour the recurrence of GIST with multiple disseminated abdominal lesions was detected, however after 2 months of treatment with imatinib mesylate, the dog achieved complete remission [120]. Tyrosine kinase inhibition by imatinib mesylate has also been demonstrated to have clinical activity on mast cell tumours in dogs [121].

Spontaneous feline oral squamous cell carcinoma (FOSCC) and head and neck squamous cell carcinoma (HNSCC) in humans share molecular markers (EGFR, VEGF, and p53), tumour biology, treatment and prognostic similarities [122, 123], with both showing comparable rates of development of metastasis (15–20%) [124]. A multimodal treatment approach is commonly used in both cats and humans with FOSCC/HNSCC, including surgery, radiation therapy and chemotherapy. Chemotherapeutic drugs routinely used in HNSCC include cisplatin and piroxicam, and novel therapeutic agents such as cetuximab, gefitinib and masitinib have also been investigated in cats [123].

### Therapeutic implications

Anti-metastatic therapeutics are a high-potential category of drugs but require complex development, which has been deprioritised by the pharmaceutical industry due to late-stage failures in clinical development, despite extensive evidence in preclinical models [125]. The current framework for cancer therapeutics is unfavourable towards anti-metastatic agents with a focus on clinical outcomes such as tumour shrinkage, rather than inhibition of metastasis. However, the implementation of comparative oncology strategies has already facilitated some progression in clinical metastasis.

Combination therapy remains the basis for clinical management of many types of cancers, as the use of multiple treatments may be more effective in targeting the heterogeneity within the tumour population and avoid the development of resistance. To this end, the use of a procaspase-activating compound 1 (PAC-1) in combination with doxorubicin was evaluated in 10 companion canines with metastatic osteosarcoma or lymphoma, and found to be efficacious in 50% (3/6) of osteosarcoma patients and 100% (4/4) of lymphoma patients [126]. Since this study, a Phase I clinical trial was launched in 2017 for PAC-1 in combination with temozolomide in patients with high grade glioma after progression following standard first line therapy (ClinicalTrials.gov Identifier: NCT03332355; study due for completion June 2019).

In both humans and dogs, treatment for osteosarcoma involves surgery to remove primary tumours and on occasion distant metastasis, combined with neoadjuvant and/or adjuvant chemotherapy [127, 128]. The similarities in disease and progression between humans and dogs, alongside utilising the compressed life-span of the companion canines, offers an opportunity to quickly identify anti-metastatic therapeutics in veterinary trials that can be used to inform new treatment options for human patients. There are several examples of where this opportunity has been harnessed. Promising results from a randomized study [129] using the immunomodulator mifamurtide (Liposomal-Muramyl TriPeptide-PhosphatidylEthanolamine: L-MTP-PE) on dogs with spontaneous osteosarcoma, in addition to subsequent studies, prompted clinical studies with this drug in humans [130]. A Phase II trial using L-MTP-PE in patients with osteosarcoma and synchronous or metachronous lung metastases concluded that there was evidence for a biological effect of L-MTP-PE on osteosarcoma lung metastases [131]. Another example is with the use of HER2 immunotherapy. A phase I dose escalation clinical trial in dogs with osteosarcoma found that ADXS31-164 (*Listeria* expressing a chimeric human HER2/neu fusion protein) administered in the setting of minimal residual disease could induce HER2/neu-specific immunity and may reduce the incidence of metastatic disease and prolong overall survival [132]. Following from this success, Advaxis recently licensed (at the end of 2018) ADXS-HER2 for evaluation (clinical trials) in the treatment of osteosarcoma in human patients. Excitingly, to addresses the current limitations in the drug discovery and development process for anti-metastatic therapeutics, Vuja De Sciences and Ethos Veterinary Health announced (at the end of 2018) they would be collaborating to empower metastasis-focused drug development, starting with osteosarcoma.

## Conclusion

The use of non-laboratory bred animals (‘metastasis in the wild’), may present its challenges but offers a more clinically-relevant model than the traditional laboratory mouse and provides a unique opportunity for clinical and experimental research that would benefit both humans and animals. Spontaneous tumours in non-laboratory animals represent relevant models for human cancer and metastasis for many reasons. A key importance is that similar to the human population, non-laboratory animals are a genetically heterogeneous population, as are the cancers and metastases they develop. In addition, they are typically larger in size and have a longer life-span than laboratory mice, and are affected by the same environmental stimuli (pollutants, infections) and disease states (diabetes, obesity, stress) that face humans. Furthermore, recent advancements in immunotherapy make the immune system a very contemporary topic in targeting metastasis, but the complexity of the human immune system can be difficult to mimic in a laboratory setting. Often kept in a controlled environment, laboratory animals exhibit differences in pathogen and microbial exposure which will elicit different immune responses, not necessarily representative of a human cancer patient. Moreover, in human-derived experimental metastasis models, immune-deficient murine hosts are required to avoid rejection of the cancer cells, further removing the set-up from relevance to the clinical setting.

In non-domestic animals, there exists the opportunity to observe the course of metastatic development and progression in the absence of therapeutic intervention (rarely achievable in human studies), to allow research into the fundamental biology driving these processes. In domestic animals, particularly companion animals, there is generally a strong emotional attachment to the animal, which translates to a high level of compliance for drug trials and attending follow up appointments, thus affording the opportunity to test new therapeutic regimes (again, rarely achievable in human studies). Thus with the number of surplus tissues, compliance to necropsy, cross-species immunoreactivity, comparable histopathological analysis and diagnosis, in addition to the option of treated or non-treated individuals, veterinary-seen animals are an economical, under-used resource in metastasis research. Each patient, whether human or animal, holds an exclusive ability to individually contribute to experimental and clinical metastasis research, and initiatives to integrate human and veterinary oncology research programs (such as the National Cancer Institute’s Center for Cancer Research (CCR) “Comparative Oncology Program”) will further contribute to expanding our understanding of metastasis and the development of therapeutic opportunities.
